# Supplementary material for: Structural basis for centromere maintenance by Drosophila CENP‐A chaperone CAL1
Source: EMBO J. 2020 Mar 5;39(7):e103234. doi: 10.15252/embj.2019103234 (PMC7110144; doi:10.15252/embj.2019103234)
Supplement: Supplementary file 1 — Appendix [file EMBJ-39-e103234-s001.pdf]

TABLE OF CONTENTS

Appendix Supplementary Methods

Purification of histones

LC-MS/MS analysis

Appendix Supplementary Methods

Purification of histones

To purify from inclusion bodies, histones were expressed in *E. coli* BL21 (*DE3*) pLysS cells grown in 2 L of LB media at 37°C until O.D 0.6. Cells were induced with IPTG to a final concentration of 0.2 mM at 37°C for 3-4 hours before harvesting by centrifugation at 4000 x g for 10 min. Pellets were resuspended in 50 mM Tris-HCl pH 8, 100 mM NaCl, 1 mM EDTA, 1 mM benzamidine and 5 mM βME and snap frozen in liquid nitrogen. After two rounds of freeze thaw cycles the cells were further lysed by sonication and any soluble proteins were separated by centrifugation at 22,000 rpm for 1 h at 4°C. The insoluble pellets were washed twice with 50 mM Tris-HCl pH 8, 100 mM NaCl, 1 mM EDTA, 1 mM benzamidine, 5 mM βME and 1% triton then twice with 50 mM Tris-HCl pH 8, 100 mM NaCl, 1 mM EDTA, 1 mM benzamidine and 5 mM βME using a glass homogeniser and pelleting by centrifugation at 22,000 rpm for 10 min at 4°C in between.

Pellets were then left to soak with 500 µl of DMSO for 15 min before resuspending in 20 mM Tris-HCl pH 7.5, 7 M guanidine HCl, 10 mM DTT. After brief sonication, samples were left rotating at RT for 2 h, the unfolded protein was then recovered by centrifugation at RT for 20 min at 22,000 rpm.

Next the histone was dialysed twice for 2 h and once overnight against 500 ml of a buffer containing 10 mM Tris pH 8, 100 mM NaCl, 7 M urea, 1 mM EDTA and 5 mM βME. Proteins were then centrifuged at 4°C at 22,000 rpm for 30 min, before briefly sonicating then passing through a Millex syringe filter (Millipore). Proteins where then further purified using ion exchange in such a way that samples were passed first through a HiTrap® Q HP column (GE Healthcare) equilibrated with dialysis buffer, then a HiTrap® SP HP column. Protein bound to the HiTrap® SP HP was eluted using a gradient of 10 mM Tris-HCl pH 8, 1 M NaCl, 7 M urea, 1 mM EDTA and 5 mM βME. After analysis by SDS-PAGE, appropriate fractions were pooled and dialysed twice for 2 h and once overnight against 2 L ddH_2O and 5 mM βME. The concentration of the histones was measured by Bradford assay and then proteins were lyophilised for storage.

LC-MS/MS analysis

LC-MS/MS analysis was performed using an Orbitrap Fusion Lumos (Thermo Fisher Scientific) with a "high/high" acquisition strategy. The peptide separation was carried out on an EASY-Spray column (50 cm × 75 μm i.d., PepMap C18, 2 μm particles, 100 Å pore size, Thermo Fisher Scientific). Mobile phase A consisted of water and 0.1% v/v formic acid. Mobile phase B consisted of 80% v/v acetonitrile and 0.1% v/v formic acid. Peptides were loaded at a flow rate of 0.3 μl/min and eluted at 0.2 μl/min using a linear gradient going from 2% mobile phase B to 40% mobile phase B over 109 followed by a linear increase from 40% to 95% mobile phase B in 11 min. The eluted peptides were directly introduced into the mass spectrometer. MS data were acquired in the data-dependent mode with a 3 s acquisition cycle. Precursor spectra were recorded in the Orbitrap with a resolution of 120,000. The ions with a precursor charge state between 3+ and 8+ were isolated with a window size of 1.6 m/z and fragmented using high-energy collision dissociation (HCD) with a collision energy of 30. The fragmentation spectra were recorded in the Orbitrap with a resolution of 15,000. Dynamic exclusion was enabled with single repeat count and 60 s exclusion duration. The mass spectrometric raw files were processed into peak lists using ProteoWizard (version 3.0.19288) (Kessner, Chambers et al., 2008), and cross-linked peptides were matched to spectra using Xi software (version 1.7.754.RC1) (Mendes, Fischer et al., 2018) with in-search assignment of monoisotopic peaks (Lenz, Giese et al., 2018). Search parameters were MS accuracy, 3 ppm; MS/MS accuracy, 10ppm; enzyme, trypsin; cross-linker, EDC or BS^3; max missed cleavages, 4; missing mono-isotopic peaks, 2; fixed modification, carbamidomethylation on cysteine; variable modifications, oxidation on methionine; fragments, b and y ions with loss of H_2O, NH3 and CH_3SOH.
