# Supplementary material for: Structural basis for centromere maintenance by Drosophila CENP‐A chaperone CAL1
Source: EMBO J. 2020 Mar 5;39(7):e103234. doi: 10.15252/embj.2019103234 (PMC7110144; doi:10.15252/embj.2019103234)
Supplement: Supplementary file 2 — Expanded View Figures PDF [file EMBJ-39-e103234-s002.pdf]

Expanded View Figures

Figure EV1. CAL1 is predicted to be predominantly unstructured.

A Disordered regions of CAL1 as predicted by Disopred (http://bioinf.cs.ucl.ac.uk/psipred). Residue number on x-axis and probability of disorder on y-axis.

B Secondary structure composition and multiple sequence alignment of CAL1 N-terminus as performed using Psipred (http://bioinf.cs.ucl.ac.uk/psipred) and MUSCLE (Madeira *et al*, 2019). Numbers correspond to *Drosophila melanogaster*. *Drosophila melanogaster (D. mel), Drosophila grimshawi (D. gri), Drosophila mojavensis (D. moj), Drosophila virilis (D. vir), Drosophila persimilis (D. per), Drosophila pseudoobscura pseudoobscura (D. pse), Drosophila ananassae (D. ana), Drosophila erecta (D. ere), Drosophila yakuba (D. yak) and Drosophila simulans (D. sim).*

C SEC-MALS analysis of His-CAL1$_{1–160}$–CENP-A$_{101–225}$–H4 and His-CAL1$_{1–160}$–CENP-A$_{144–225}$–H4. Absorption at 280 nm (mAU, left *y*-axis) and molecular mass (kDa, right *y*-axis) are plotted against elution volume (ml, *x*-axis). Measured MW and the calculated subunit stoichiometry based on the predicted MW of different subunit compositions. Samples were analysed using Superdex 200 increase 10/300 in 50 mM HEPES pH 8.0, 2 M NaCl and 1 mM TCEP.

 © 2020 The Authors

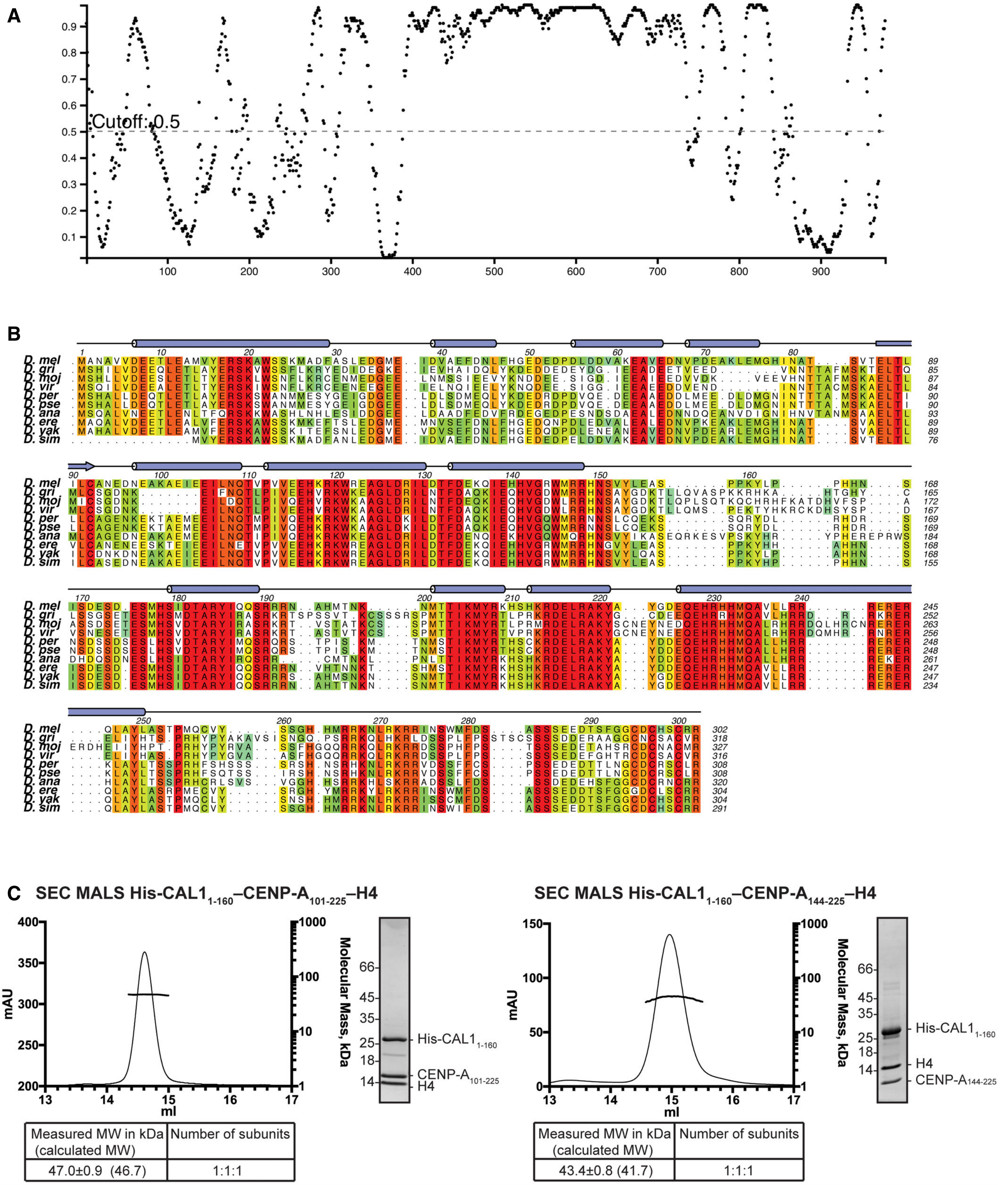
A
Cutoff: 0.5
B
D. mel
D. gri
D. moj
D. vir
D. per
D. pse
D. ana
D. ere
D. yak
D. sim
C
SEC MALS His-CAL1 1-160–CENP-A 101-225–H4
mAU
ml
Molecular Mass, kDa
His-CAL1 1-160
CENP-A 101-225
H4
Measured MW in kDa (calculated MW) | Number of subunits
47.0±0.9 (46.7) | 1:1:1
SEC MALS His-CAL1 1-160–CENP-A 144-225–H4
His-CAL1 1-160
H4
CENP-A 144-225
Measured MW in kDa (calculated MW) | Number of subunits
43.4±0.8 (41.7) | 1:1:1

Figure EV1.

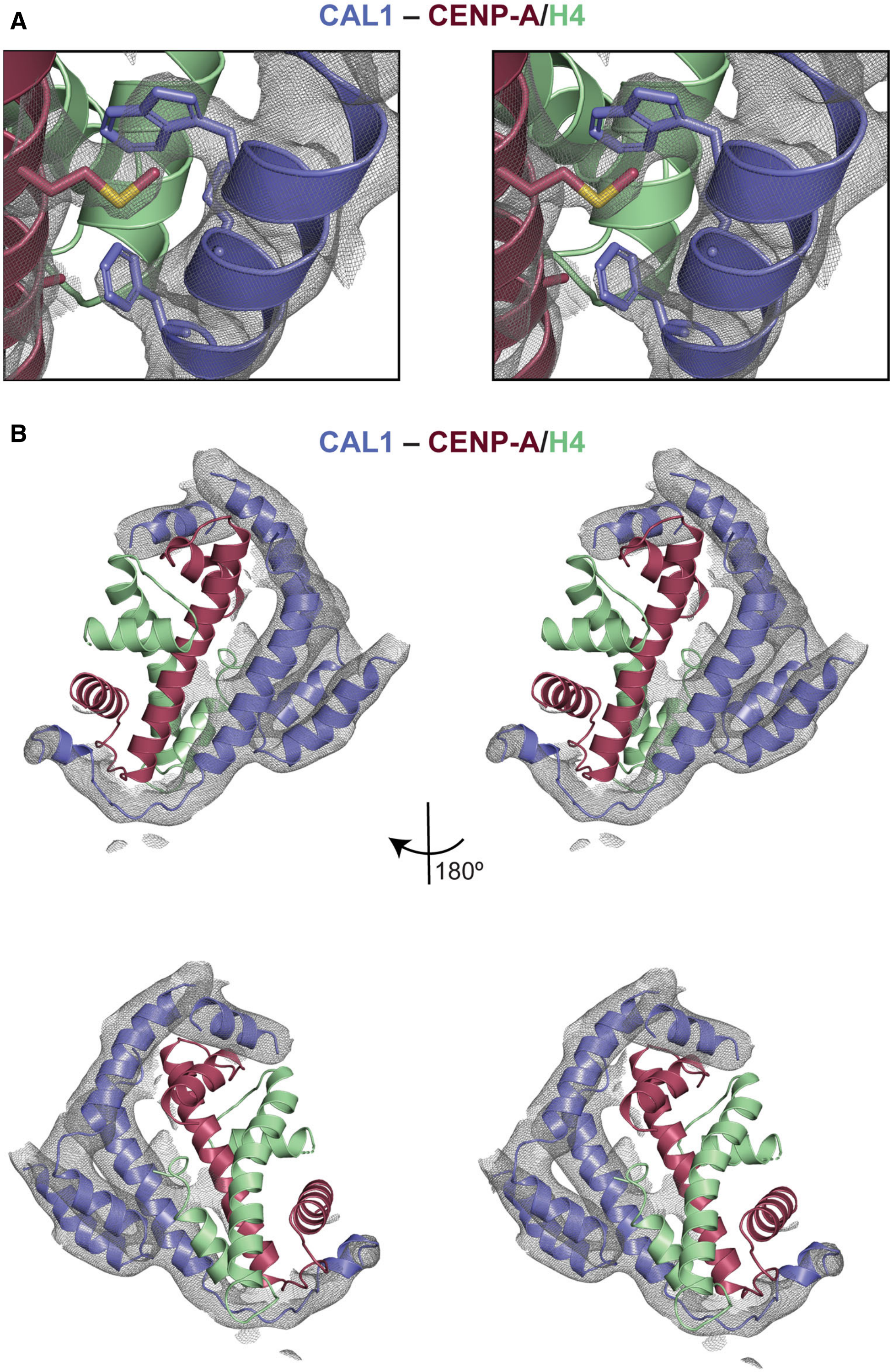

Figure EV2. Stereo images of the electron density maps and corresponding final models of CAL1–CENP-A/H4 from crystal form I and II.

A $2F_o$–F_c electron density map contoured at 1 sigma for the vicinity of CAL1 residues W22 and F29 for crystal form I. CENP-A in maroon and H4 in green.
B $2F_o$–F_c electron density map contoured at 1 sigma for CAL1 bound to CENP-A/H4 for crystal form II. CAL1 is shown in blue, CENP-A in maroon and H4 in green.

 © 2020 The Authors

Figure EV3. Intra- and intermolecular contacts identified between CAL1 and CENP-A/H4 using cross-linking mass spectrometry (CLMS).

A SDS–PAGE analysis of His-$CAL1_{1–160}$-CENP-$A_{101–225}$-H4 cross-linked with both EDC (left) and BS^3 (right) cross-linker.

B Linkage map showing the sequence position and cross-linked residue pairs between His-$CAL1_{1–160}$, CENP-$A_{101–225}$ and H4. Cross-linked samples were resolved with SDS–PAGE and then analysed by MS. CAL1 is shown in blue, CENP-A in maroon and H4 in green. Solid lines represent EDC cross-links, while dashed lines show BS^3 cross-links.

C High-resolution representative fragmentation spectra displayed using XiSpec (Ref: PMID: 29741719) for cross-linked peptides seen between $CAL1_{1–160}$ and $CAL1_{1–160}$, $CAL1_{1–160}$ and CENP-$A_{101–225}$ and $CAL1_{1–160}$ and H4.

© 2020 The Authors

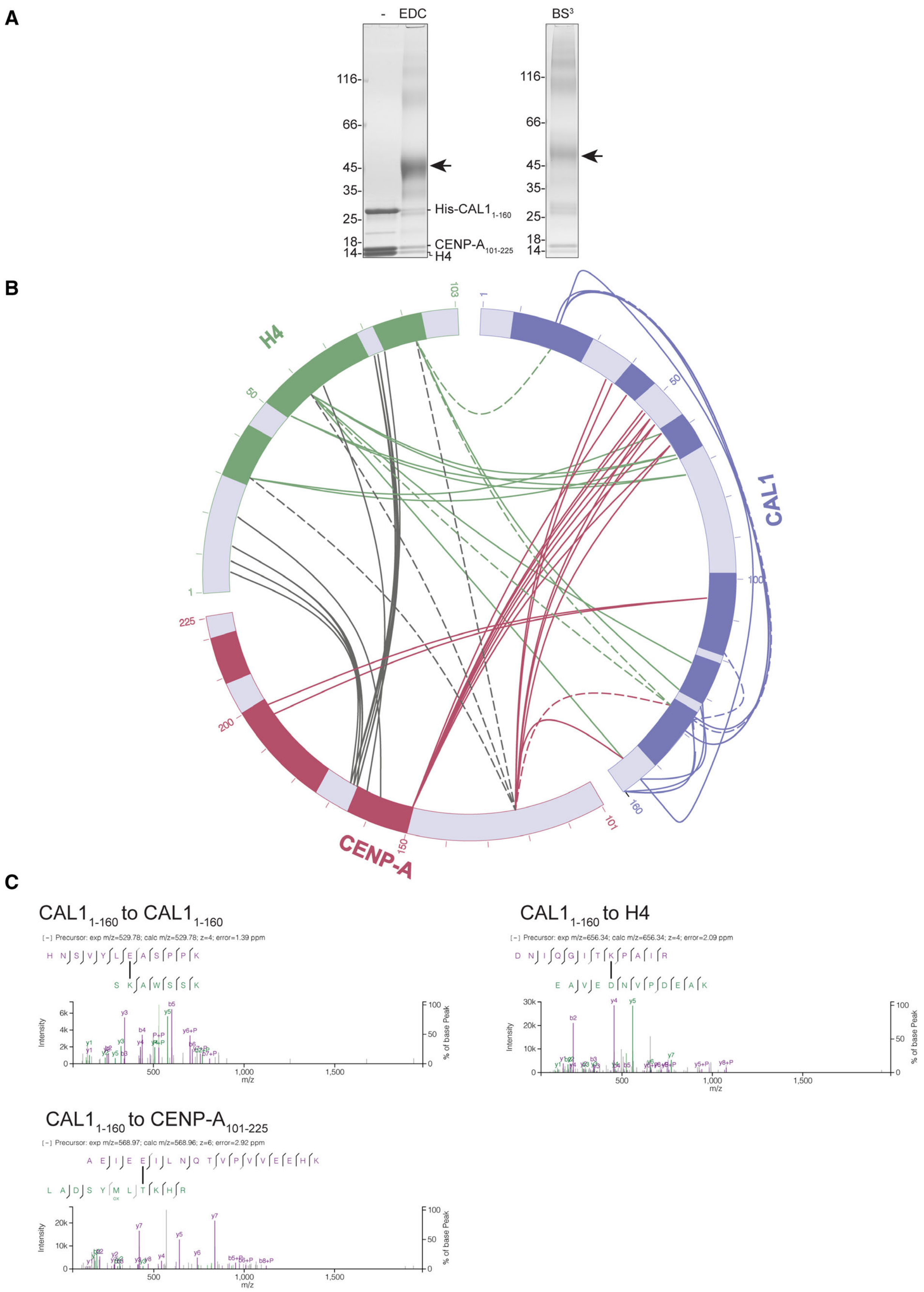

Figure EV3.

 © 2020 The Authors

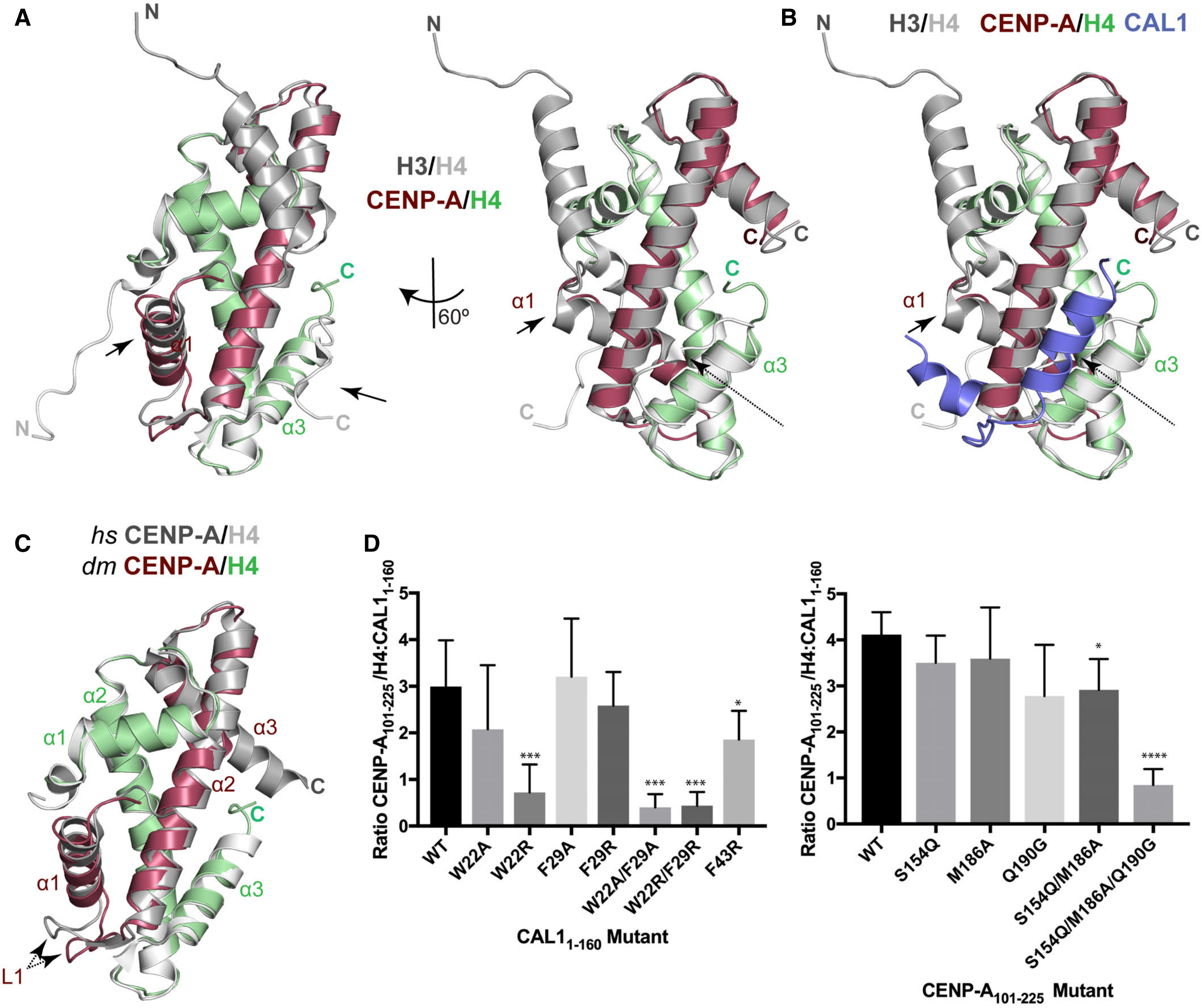

Figure EV4. Mode of H4 recognition by CENP-A by CAL1.

A Structural superposition of CENP-A/H4 with H3/H4 (Clapier *et al*, 2008). CENP-A is shown in maroon, H4 in green and H3/H4 in silver. Arrows indicate conformational changes.

B Structural superposition of CAL1–CENP-A/H4 (form I) with H3/H4 (Clapier *et al*, 2008). CAL1 is shown in blue, CENP-A in maroon, H4 in green and H3/H4 in silver. Arrows indicate conformational changes; dotted arrow highlights conformational changes in the loop regions.

C Structural superposition of *hs* CENP-A/H4 (Sekulic *et al*, 2010) with *dm* CENP-A/H4. *dm* CENP-A in maroon, *dm* H4 in green and *hs* CENP-A/H4 in silver. Dotted arrow highlights conformational changes in the loop regions.

D (left panel) Quantifications of Ni-NTA pull-down of His-$CAL1_{1\text{–}160\ WT}$ and indicated mutants with $CENP\text{-}A_{101\text{–}225}$–H4. Bar graph shows average ratio of the band intensities between $CENP\text{-}A_{101\text{–}225}$/H4 and His-$CAL1_{1\text{–}160}$ (n = 7 experiments). (right panel) Quantifications of Ni-NTA pull-down of His-$CAL1_{1\text{–}160\ WT}$ with $CENP\text{-}A_{101\text{–}225}$–H4 and indicated mutants. Bar graph shows average ratio of the band intensities between $CENP\text{-}A_{101\text{–}225}$/H4 and His-$CAL1_{1\text{–}160}$ (n = 4 experiments).

Data information: In (D), (left panel) data presented as mean ± SD of 7 experiments, P-values were calculated using a Mann–Whitney test. (right panel) Data presented as mean ± SD of 4 experiments, P-values were calculated using unpaired two-tailed t-test. (*$P < 0.05$, ***$P < 0.001$, ****$P < 0.0001$).

© 2020 The Authors

A
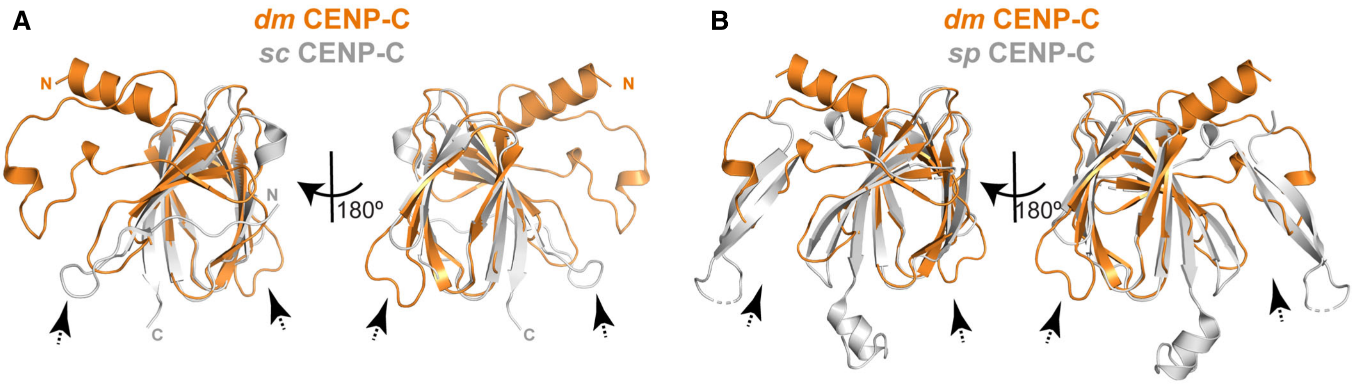

C

Species	Mass (g/mol) Calculated	Mass (g/mol) Experimental	$S^{0}_{20,w}$ (S) Computed	$S^{0}_{20,w}$ (S) Experimental
$CAL1_{841\text{-}979}$	15,391	19,111	1.48	1.46
$(CAL1_{841\text{-}979})_2$	30,782		2.02	
$CENP\text{-}C_{1264\text{-}1411}$	17,187	33,867	1.77	2.74
$(CENP\text{-}C_{1264\text{-}1411})_2$	34,374		2.96	
$(CENP\text{-}C_{1264\text{-}1411})_2$ + $CAL1_{841\text{-}979}$	49,765	48,889	3.47	3.50
$(CENP\text{-}C_{1264\text{-}1411})_2$ +$(CAL1_{841\text{-}979})_2$	65,156		3.89	

D
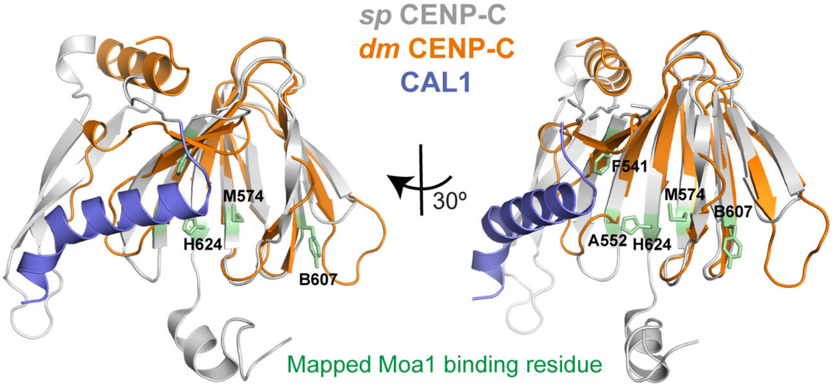

Figure EV5. CENP-C structure and binding to CAL1.

A Structural superposition of cupin domains of *dm* CENP-C and *sc* CENP-C (Mif2p) (Cohen *et al*, 2008). CENP-C is shown orange and Mif2p in silver. Dotted arrow highlights conformational changes in the loop regions.

B Structural superposition of cupin domains of *dm* CENP-C and *sp* CENP-C (Cnp3) (Chik *et al*, 2019). CENP-C is shown orange and Cnp3 in silver. Dotted arrow highlights conformational changes in the loop regions.

C Calculated and experimentally determined molecular masses and sedimentation coefficients for plausible solution states of $CAL1_{841–979}$, $CENP\text{-}C_{1264–1411}$ and complexes thereof.

D Structural superposition of CAL1 bound *dm* CENP-C cupin domain onto *sp* CENP-C cupin domain. *dm* CENP-C is shown orange, *sp* CENP-C in silver and CAL1 in blue. Amino acid residues identified to be crucial for Moa1 binding (by Chik *et al*, 2019) are shown in stick representation in green.

 © 2020 The Authors
